# Is occupational exposure to radiofrequency electromagnetic fields associated with glioma risk? An Australian population-based family case–control study

**DOI:** 10.1136/bmjopen-2025-107281

**Published:** 2026-03-12

**Authors:** Rohan Mate, Geza Benke, Sarah P Loughran, Michael J Abramson, Claire Vjadic, Michelle Turner, Maxime Turuban, Elisabeth Cardis, Ken Karipidis

**Affiliations:** 1 Public Health and Preventive Medicine, Monash University, Melbourne, Victoria, Australia; 2 ARPANSA, Yallambie, Victoria, Australia; 3 Department of Epidemiology and Preventive Medicine, Monash University, Melbourne, Victoria, Australia; 4 UNSW Medicne Kirby Institute, Sydeny, New South Wales, Australia; 5 Barcelona Institute for Global Health, Barcelona, Spain; 6 Universitat Pompeu Fabra, Barcelona, Spain; 7 Monash University, Melbourne, Victoria, Australia

**Keywords:** Case-Control Studies, Cancer, OCCUPATIONAL & INDUSTRIAL MEDICINE

## Abstract

**Abstract:**

**Objectives:**

This study investigated occupational exposure to radiofrequency electromagnetic fields (RF EMF) using two job-exposure matrices (JEMs) and risk of glioma.

**Design:**

Population-based family case–control study.

**Setting:**

Cases were recruited from participating hospitals in the Australian states of New South Wales, Queensland, Tasmania, Western Australia and Victoria between January 2013 and November 2017.

**Participants:**

The study population consisted of 467 cases of glioma and 367 family controls recruited for the Australian Genomics and Clinical Outcomes of Glioma case–control study between 2013 and 2017. Participants completed questionnaires on demographic and other information, including a detailed occupational history.

**Exposures:**

Exposure to RF EMF was estimated using both the multicountry case–control study INTEROCC JEM and the Canadian JEM (CANJEM).

**Primary outcome measures:**

ORs and 95% CIs were calculated from logistic regression models adjusted for relatedness between cases and controls, sex, age, ethnicity, education level, smoking status and alcohol consumption.

**Results:**

There was no statistically significant positive association overall for risk of glioma when applying either JEM. For the highest compared with the lowest quartile of lifetime exposure, results using the INTEROCC JEM showed an OR of 0.74 (95% CI 0.47 to 1.15) for electric fields and 0.92 (95% CI 0.58 to 1.45) for magnetic fields, while the CANJEM showed an OR of 0.85 (95% CI 0.54 to 1.32). We also did not observe associations when applying different assumptions regarding latency or time windows or with glioma grade.

**Conclusion:**

Overall, this study found no evidence of an association between occupational RF EMF exposure and glioma. Future research should focus on refining occupational RF EMF exposure assessment.

**STRENGTHS AND LIMITATIONS OF THIS STUDY**

- For cases to be included in the study, a histopathological diagnosis of any grade of glioma was required and all participants were recruited within 3 years of their diagnosis.
- The use of family-based controls resulted in a high participation rate, reducing potential non-participation bias.
- The use of two comprehensive job-exposure matrices designed to assess exposure to radiofrequency electromagnetic fields (RF EMF) is a further strength.
- Exposure misclassification is a possible weakness of this study, particularly since the RF EMF exposure prevalence in many jobs was low.

## Introduction

 Radiofrequency electromagnetic fields (RF EMF) are a type of non-ionising radiation in the frequency range of the electromagnetic spectrum between 100 kHz and 300 GHz. Exposure to sufficiently high levels of RF EMF can heat biological tissues.^1^ Exposure guidelines are produced by the International Commission on Non-Ionizing Radiation Protection (ICNIRP).^1^ No health effects of RF-EMF at lower levels have been clearly demonstrated to date; however, there remains concern about possible effects, particularly an association with brain tumours.^2^

Exposure to RF EMF in the environment is mainly from wireless communications sources and is generally low for members of the public.^3 4^ However, relatively high levels of exposure to RF EMF can occur to workers in the manufacturing, transport, medical, construction and communications industries, when work is conducted in close proximity to RF transmitting sources and/or with the use of short range communication devices.^5^,^7^ For example, measurement surveys have shown medical practitioners using diathermy are routinely exposed to RF EMF levels at 6% of the ICNIRP exposure limits, while exposures can be higher for plastic welders at 11%, manufacturing workers using induction heating at 17% and various occupations using handheld two-way radio at 94% of the exposure limits.^8^ The amount of RF EMF routinely encountered by these workers during occupational tasks is generally too low to produce significant heating or increased body temperatures that could be associated with adverse health effects.^1 5^

A limited number of previous epidemiological studies have investigated the association between occupational exposure to RF EMF and glioma, which is the most common type of primary malignant brain tumour and whose aetiology remains largely unknown.^9^,^14^ A recent WHO-commissioned systematic review of the impact of RF EMF on cancer found occupational exposure to RF EMF was not associated with glioma risk, but the certainty in the evidence was low as it was based on only three studies.^15^ Subsequent to the WHO systematic review, a large-scale case–control study by Turuban *et al*^7^ has been published, also concluding there was no clear association between occupational RF EMF and glioma in a seven-country study. However, the study did report some elevated ORs in the highest RF EMF exposure category when estimating exposure for specific recent time periods before diagnosis or recruitment. Although these elevated ORs may indicate a tumour promotion effect, they could also be due to bias or chance from multiple comparison tests.^7^ Further examination of this topic was required to fully understand the potential impact of occupational RF EMF exposure on glioma.

A key concern across all the research into occupational exposures and health outcomes is the quality of the RF EMF exposure assessment.^5^ Previous studies have often used job titles and distance to a source as exposure surrogates or qualitative measures of exposure assessment such as expert assessment.^9 13 16^ Other studies have estimated exposure levels based on spot measurements, information from the literature or through source-based estimates from measurement data.^11 13^ These types of exposure assessment methods do not necessarily provide accurate estimates and could lead to non-differential misclassification.^17^

More recent studies have used job-exposure matrices (JEMs) as a method of assessing retrospective occupational exposure. A JEM assigns exposure estimates (such as exposure level and probability of exposure) to job titles.^18^ However, until recently, there have been few JEMs that have assigned RF EMF exposure to job titles, and of these, only a small number of job titles were assigned exposures.^19 20^ Two more recent JEMs, the multinational INTEROCC JEM and the Canadian JEM (CANJEM), have assigned RF EMF exposure to more job titles compared with previous JEMs and therefore provide greater opportunity for assessing potential health effects associated with occupational RF EMF exposure in population-based studies.^5 21^

The aim of our study was to use improved RF EMF exposure assessment tools that are now available to assess whether occupational exposure to RF EMF is associated with glioma. We applied both the INTEROCC JEM and CANJEM to the work history data from the Australian Genomics and Clinical Outcomes of Glioma (AGOG) family case–control study.^22^

## Methods

### Study design and participants

Information on the AGOG study population has been described previously, including subject characteristics and exclusions.^22 23^ Briefly, cases were adults aged between 18 and 85 years with a histopathological diagnosis of glioma and recruited within 3 years of diagnosis from participating hospitals in the Australian states of New South Wales, Queensland, Tasmania, Western Australia and Victoria between January 2013 and November 2017. Information on cancer grade and morphology, cancer site and time from diagnoses was collected from clinical case records. The AGOG controls were partners and siblings of cases who had not been diagnosed with glioma and were able to complete the questionnaires themselves in English.^22 23^ Persons who were non-residents of Australia, unable to provide written consent (or deemed to be significantly cognitively impaired) or unable to complete the questionnaire in English were excluded.^22 23^

Participants were asked to complete a questionnaire about demographic characteristics, education, income, smoking status, including if they were a current or former smoker and their amount of alcohol consumption. In addition, each subject completed a lifetime calendar with a detailed occupational history including each job title, employer, industry, start and finish years, number of hours worked per day and number of days worked per week. Of the 521 cases and 381 controls originally recruited, 54 cases and 14 controls were excluded, because they did not report their occupational histories, leaving data from 467 cases and 367 controls for this analysis.

### Exposure assessment

Exposure to RF EMF was estimated using both the INTEROCC JEM and CANJEM. The INTEROCC JEM assigned occupational RF EMF exposures based on various published data including measurements and RF source information collected between 1974 and 2013 for 468 job titles.^3^ The INTEROCC JEM was based on quantitative measurements of occupational sources of RF EMF, self-reported task information from INTEROCC study participants and exposure coded into International Standard Classification of Occupations (ISCO) 88 four-digit codes.^14^ The INTEROCC JEM provided estimates for both the electric field (E-field) and the magnetic field (H-field) which were calculated as squared ratios of the time weighted average (TWA) exposure levels and the ICNIRP reference levels. The resulting metric this JEM provided was unitless and could be described as an exposure intensity rating, together with an estimate of the prevalence of exposure in each job title. A detailed description of the INTEROCC JEM has been previously described.^5^

CANJEM was based on a pooled dataset of 8912 subjects from four case–control studies conducted between 1979 and 2004. Using this dataset, occupational exposures to 294 agents for 119 occupations were classified and coded into ISCO-68. CANJEM was subsequently recoded into ISCO-88 codes. CANJEM was based on expert assessment with exposure to RF EMF being rated as low, medium and high and expressed as an intensity rating of 1, 2 and 3, respectively. An exposure prevalence represented by a proportion from 0 to 1 was also applied.^21^ Unlike the INTEROCC JEM, CANJEM only provided estimates for RF EMF and not the E-field and H-field components separately. A detailed explanation of CANJEM has been previously published.^21^

All jobs held by each subject were manually allocated an ISCO-88 occupational code by Odutola *et al*^24^ which were then mapped in the current study to the RF EMF exposures according to the INTEROCC JEM and CANJEM. Occupational exposure for each subject was calculated by multiplying the duration of employment (in ‘years’) in each work history record by the corresponding entry of the JEM (intensity rating) and aggregating across the total work history to obtain a cumulative estimate of exposure in arbitrary units of ‘intensity rating years’. The calculation of cumulative exposure for each participant is shown by the following formula:


$$Cumulative\;exposure=\sum_{j=1}^{Total\;number\;of\;job}Duration_{j}\times Intensity\;rating_{j}$$

The researchers were blinded to the case or control status of the participants when applying the exposure metrics.

### Statistical analyses

We assessed lifetime cumulative exposure as a continuous variable by log_e_ transforming the cumulative exposure distribution. To account for zero values, we added an offset of +1 to all the values, which was the minimum offset that could be used to preserve exposure values ≥0, while keeping distortion of the distribution at lower levels at a minimum. We also assessed four levels of exposure by dividing the cumulative lifetime exposure distribution (including the unexposed) into quartiles and using the lowest quartile as the reference group. For both JEMs, the ORs and 95% CIs calculated were adjusted for relatedness between cases and controls, sex, age, ethnicity, education, smoking status and alcohol consumption, using multivariate logistic regression. The variance–covariance matrix of the estimators cluster option was applied in the models to account for potential correlations between cases related to or cohabiting with controls.^24^

To account for possible latency of the effect of RF EMF on the development of glioma, ORs and 95% CIs were also calculated for 2-year, 5-year and 10-year lag periods prior to diagnosis for cases or recruitment for controls. ORs were also calculated for specific time windows; when applying time windows, only exposures within the specific time frame were assessed for each participant.^7^ Time windows assessed whether recent RF EMF exposures rather than cumulative total lifetime exposure to RF EMF had a greater impact on cancer risk.^13^ The time windows applied were for exposure in the last 1–4 years or 5–9 years before diagnosis or recruitment. Because time windows did not include many exposed subjects, the cumulative exposure was broken into higher percentiles including <75%, 75%–90% and >90% with the <75th percentile being used as the reference group.^7^ Furthermore, ORs were also calculated for glioma grades 1–4 individually.

The data were also tested for the impact of missing data for age, alcohol use and education variables (online supplemental appendix table 4). A sensitivity analysis was done to determine the impact that genetically related controls had on the outcome of the logistic regression model. Another sensitivity analysis was conducted to examine how exposure prevalence impacted the data when applying the INTEROCC JEM. This was done by only assigning exposure estimates for jobs with RF exposure prevalences above the median prevalence of exposure among all exposed jobs.^7^ All analyses were conducted using Stata V.17 (StataCorp, College Station, Texas, USA).^25^

### Patient and Public Involvement Statement

No patients or community representatives were involved in the design, conduct, reporting or dissemination plans of this research.

## Results

The characteristics of 467 cases of glioma and 367 controls are shown in table 1. The sex distribution was not even, with more male cases (63%) compared with controls (38%). However, the mean age of the subjects was similar between cases (54 years with a SD of 14 years) and controls (53 years with a SD of 13 years). Most cases (62%) and controls (63%) received education after high school either through university or other technical training. Cases were more likely to have ever smoked (49%) compared with controls (39%). Almost all subjects were Caucasian (97% cases, 98% controls). The mean number of years worked was also similar for cases (29±SD 19 years) and controls (27±17 years).

**Table 1:** Characteristics of included participants

Characteristics	No of cases	No of controls	P value
	n=467 (% of cases)	n=367 (% of controls)	
Sex			
Male	292 (63%)	138 (38%)	
Female	175 (37%)	229 (62%)	<0.01
Highest attained education			
High school	152 (32%)	119 (32%)	
Higher education	288 (62%)	233 (63%)	
Other	8 (2%)	6 (2%)	
Missing	19 (4%)	9 (3%)	0.63
Ethnicity			
Caucasian	453 (97%)	361 (98%)	
Other	1 (<1%)	0	
Missing	13 (3%)	6 (2%)	0.37
Smoking status			
Never	227 (49%)	216 (59%)	
Ever	227 (49%)	144 (39%)	
Missing	13 (2%)	7 (2%)	0.01
Alcohol use			
Ever	397 (85%)	328 (89%)	
Never	55 (12%)	32 (9%)	
Missing	15 (3%)	7 (2%)	0.16

Most cases and controls were occupationally exposed to RF EMF when applying the two JEMs; 95% of cases and 97% of controls for the INTEROCC JEM E-field, and 94% of cases and 95% of controls for the INTEROCC JEM H-field. When assigning exposure estimates for jobs with RF exposure prevalences above the median prevalence of exposure among all exposed jobs, 27% of cases and 22% of controls were exposed to the E-field, while 36% of cases and 31% of controls were exposed to H-field. When applying CANJEM, 78% of cases and 81% of controls were exposed.

The cumulative RF EMF exposure for the entire work history of all the subjects was highly skewed (online supplemental appendix figures 1–3). The median cumulative exposure when applying the INTEROCC JEM (E-field) was 20.4 intensity rating years, compared with the INTEROCC JEM (H-field) which had a median cumulative exposure of 3.4 intensity rating years. CANJEM had a median cumulative exposure to RF EMF of 1.3 intensity rating years, noting that the intensity rating measure is not directly comparable between CANJEM and the INTEROCC JEM. The most exposed occupations that were common across both JEMs for cases and controls were printing machine operators, engineering technicians, operations and executive managers, shop keepers and crane operators (exposure estimates of the 10 highest exposed occupations in the two JEMs are shown in online supplemental appendix table 10). In the top 25% of exposed subjects using the INTEROCC JEM (E-field), there were 105 unique occupations represented, compared with 107 for the INTEROCC JEM (H-field). There were 55 occupations represented in the top 25% of exposed cases using the CANJEM.

**Figure 1:**
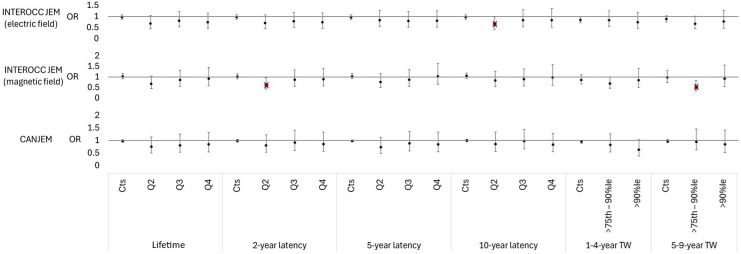
Risk of glioma relative to exposure to RF EMF based on exposure estimates from the INTEROCC JEM (E-field), INTEROCC JEM (H-field) and CANJEM. ORs adjusted for relatedness between cases and controls, sex, age, ethnicity, education level, smoking status and alcohol consumption. ORs for reference groups not displayed and can be found in the online supplemental tables S1–S3. Statistically significant outcomes in red with crosses (x). CANJEM, Canadian Job-Exposure Matrix; Cts, lifetime cumulative exposure; E-field, electric field; H-field, magnetic field; JEM, job-exposure matrix; %Ie, percentile; Q2, second quartile; Q3, third quartile; Q4, fourth quartile; RF EMF, radiofrequency electromagnetic field; TW, time window.

The ORs for glioma for lifetime, 2-year, 5-year and 10-year lag, and time window periods for >1–4 and >5–9 years of exposure are shown in figure 1 (more detailed results are presented in online supplemental appendix tables 1–3). No statistically significant elevated ORs were observed for any of the exposure parameters tested. However, there were three statistically significant inverse ORs observed when applying the INTEROCC JEM, with no clear pattern with respect to the E-field or H-field component, latency or time window. When only assigning exposure estimates for jobs with RF exposure prevalences above the median prevalence of exposure among all exposed jobs in the INTEROCC JEM, no statistically significant elevated or reduced ORs were observed (online supplemental appendix tables 8,9). There were also no statistically significant associations observed for glioma grades 1–4 when applying any of the JEMs (online supplemental appendix tables 1–3).

The sensitivity analyses showed no significant impact on the risk estimates when missing age, alcohol and education data were imputed (online supplemental appendix table 4); however, CIs did reduce in width. The removal of genetically related controls also did not impact on the results (online supplemental appendix tables 5–7).

## Discussion

In this study, we investigated the relationship between occupational exposures to RF EMF and the risk of glioma by applying two JEMs for exposure assessment. No elevated ORs were observed when the participants’ entire work histories were examined, or when latency or time windows were applied. We also did not observe an association with grade of glioma.

However, three statistically significant reduced ORs were observed with no clear pattern. We are unaware of a possible mechanism for a protective effect of RF EMF on cancer risk,^7^ and given the multiple comparisons conducted in this study, we could not exclude that these findings arose by chance. Previous studies examining occupational RF EMF exposure and brain cancer, including those included in the recent WHO commissioned systematic review,^15^ have also generally not found increased ORs in relation to occupational RF EMF exposure and glioma. In an Australian study of 414 glioma cases, Karipidis *et al*^11^ found no positive associations. However, that study only estimated RF EMF exposure for four occupations. A more recent multinational study of 1943 glioma cases from INTEROCC by Vila *et al*^14^ applied a RF source-exposure matrix and generally also reported no positive associations overall. A further analysis of the INTEROCC study by Turuban *et al*^7^ using the INTEROCC RF JEM (built on the RF source-exposure matrix) found similar results.

A strength of our study was that the AGOG case–control data were of high quality. For cases to be included in the study, a histopathological diagnosis of high to low grade glioma was required and all participants were recruited within 3 years of their diagnosis. The use of family-based controls resulted in a high participation rate, as family members had strong motivation to participate.^22^ This reduced the potential non-participation bias associated with traditional case–control studies.^26^ The use of family controls further mitigated the risk of the healthy worker effect, a bias more typical of cohort studies, but still relevant in occupational case–control studies. By using partners and siblings, the cases and controls were more likely to share socioeconomic backgrounds, lifestyles and health-seeking behaviours than general population controls. Furthermore, the characteristic rapid onset of glioma means cases generally lack the prolonged prediagnostic ‘unhealthy’ phase that typically drives the healthy worker effect in other chronic diseases.

The use of two different JEMs designed to assess exposure to RF EMF was a further strength of the present study. Both the INTEROCC JEM and CANJEM were developed using data from similar time periods.^5 21^ However, the INTEROCC RF JEM is a quantitative JEM, while CANJEM is qualitative.^21^ The INTEROCC JEM was based on published quantitative measurements and self-reported task information to assign exposure levels and prevalences.^5^ INTEROCC assigned exposure estimates to many occupations; however, a large proportion of these were based on a small number of measurements or other information.^27^ In contrast, CANJEM was based on expert assessment to assign exposure categories.^21^ CANJEM assigned exposure estimates to a much lower number of occupations compared with the INTEROCC JEM (119 vs 468). Application of the two JEMs showed different median cumulative exposures due to the variation in the exposure intensity rating, noting that CANJEM was based on expert assessment and only applied three exposure contrasts. Furthermore, the INTEROCC JEM had different median cumulative exposures for the E-field and H-field components due to how the intensity rating was calculated as a ratio of the TWA exposure and the ICNIRP reference levels.^3^

When comparing the cumulative exposure distribution, the INTEROCC JEM (E-field component) had a wider spread suggesting more contrast between low and high exposures and therefore less chance that misclassification would shift workers across quartiles. In contrast, the INTEROCC JEM (H-field component) and CANJEM produced much more compressed exposure ranges, meaning that even minor inaccuracies could move individuals between categories and increase the likelihood of non- differential misclassification. Despite these differences in exposure prevalence, intensity assignment and contrast, the two JEMs yielded risk estimates close to unity, suggesting that either the true association was weak or that misclassification was substantial across the JEMs, sufficient to attenuate any underlying exposure–response relationship. Using these two JEMs in combination allowed for comparison of results. It also resulted in greater confidence in the overall outcomes of the study as both JEMs, when applied to the AGOG data, had similar results. No statistically significant elevated ORs between RF EMF exposure and glioma were observed.

Another strength of our study was the similarity in the work histories of the participants compared with the data from which the two JEMs were developed. Approximately two thirds of the INTEROCC exposure data was from measurements taken between the 1990s and 2000s and approximately one third from the 1970s to the 1980s.^13^ However, for CANJEM, the majority of exposure information used for expert assessment was older.^28^ Most of the AGOG data were from a similar year range, with 79% of the AGOG job histories from the same year range as the exposure measurements used in the creation of the INTEROCC JEM; noting that occupational histories from participants from the Australian component of the INTEROCC study also contributed to development of the INTEROCC JEM.

A probable weakness of our study, like any case–control study, is the prospect of recall bias. It is possible that our results could have been affected by recall differences between cases and controls, particularly with regard to the impact of glioma on cases’ ability to remember details of past jobs. Since subjects were generally unaware of RF EMF exposure levels, any recall bias was probably small.

Another weakness in our study was the imbalance in the case–control numbers, thus reducing the precision of the risk estimates. Furthermore, the relatively small number of subjects included also limited the analysis, which was likely underpowered and unable to detect modest associations.

There was also a sex imbalance between cases and controls in our study, with fewer male controls compared with cases. Many occupations with RF EMF exposure are typically more common in males; however, we did not observe any increased ORs. Another limitation is that almost all subjects were Caucasian, which may affect the generalisability of the results.

Although the JEMs used in this study have improved the exposure assessment from previous research, exposure misclassification was still possible, particularly since the exposure levels and prevalences assigned differed between the two JEMs. Differences in exposure prevalence and intensity estimates between the two JEMs could lead to apparent discrepancies in glioma risk estimates. The misclassification was most likely non-differential, biasing the results towards the null. However, the results were similar between the two JEMs. Furthermore, the use of family controls introduced a possible correlation of exposures between cases and spouses or sibling controls, as they were more likely to live together, share similar lifestyles and have similar careers resulting in ORs for these case/control pairs to be biased towards the null.^26 29^ When only unrelated controls were used in the sensitivity analysis, the CIs widened.

The possibility of confounding with other occupational exposures was not considered in our analysis and remains a limitation. Subjects in this study may have been exposed to a wide variety of agents, some of which may have been closely correlated with their RF EMF exposure. There is debate over what occupational exposures are possibly associated with both RF EMF and glioma. Previously, both occupational solvents and ionising radiation have been suggested as potential causes of glioma.^16 30^ Associations have been reported between glioma and occupational exposures to arsenic, mercury and petroleum products.^31^ However, a recent study that examined 11 different occupational solvents, including gasoline, using the INTEROCC data set found no clear indication that solvent exposure was associated with an increased glioma risk.^32^ Another study of occupational ionising radiation exposure and glioma also found no increased risk of glioma even in the high exposure categories.^11^ A further study of occupational exposure to metal fumes during welding also did not find an association with glioma.^33^ Overall, the possibility of residual confounding remained.

## Conclusion

The current analysis found no evidence of elevated ORs between occupational RF EMF exposure and glioma when applying either the INTEROCC JEM or CANJEM. Future research should focus on refining occupational RF EMF exposure assessment including more accurately describing the highest exposed occupations and exposure levels and sources over time. Migault *et al*^5^ have specifically identified that current exposure estimates in the INTEROCC JEM are based on limited data. Future research should include direct measurement in diverse settings and countries.^24^

## Supplementary material

10.1136/bmjopen-2025-107281 online supplemental file 1

## Data Availability

Data may be obtained from a third party and are not publicly available.
