# Supplementary material for: Is occupational exposure to radiofrequency electromagnetic fields associated with glioma risk? An Australian population-based family case–control study
Source: BMJ Open. 2026 Mar 12;16(3):e107281. doi: 10.1136/bmjopen-2025-107281 (PMC12983896; doi:10.1136/bmjopen-2025-107281)
Supplement: online supplemental file 1 [file bmjopen-16-3-s001.docx]

**Appendix 1**

**
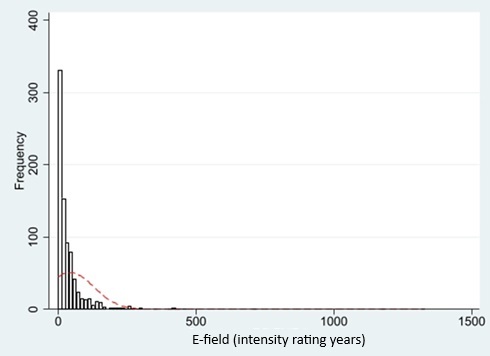
**

Figure S1: Distribution of the cumulative E-field exposure from the INTEROCC JEM (electric field) for the entire work history of 467 glioma cases and 367 controls.
Normal distribution represented by a red dotted line

**
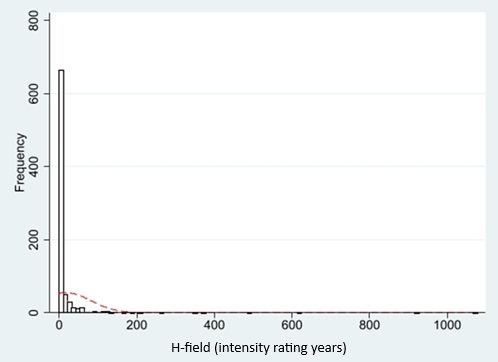
**

Figure S2: Distribution of the cumulative H-field exposure from the INTEROCC JEM (magnetic field) for the entire work history of 467 glioma cases and 367 controls.
Normal distribution represented by a red dotted line

**
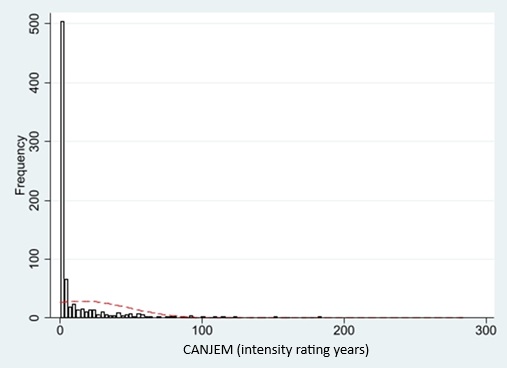
**

Figure S3: Distribution of the cumulative exposure from CANJEM for the entire work history of 467 glioma cases and 367 controls.
Normal distribution represented by a red dotted line

Table S1: Risk of glioma relative to exposure to RF EMF based on exposure estimates from the INTEROCC JEM (electric field)

|  | | RF EMF exposure (Intensity rating years) | Cases | Controls | OR† | 95% CI |
| --- | --- | --- | --- | --- | --- | --- |
| INTEROCC job exposure matrix (electric field)* | | | | | | |
| Lifetime | Continuous (log Intensity rating years) | | 467 | 367 | 0.97 | 0.87-1.08 |
| 1^st^ quartile | <5.8 | | 119 | 89 | 1.00 |  |
| 2^nd^ quartile | ≥5.8-<20 | | 107 | 102 | 0.68 | 0.44-1.04 |
| 3^rd^ quartile | ≥20-<46 | | 118 | 91 | 0.80 | 0.53-1.23 |
| 4^th^ quartile | ≥46 | | 123 | 85 | 0.74 | 0.47-1.15 |
|  | | | | | | |
| 2-year latency | Continuous (log Intensity rating years) | | 467 | 367 | 0.97 | 0.87-1.08 |
| 1^st^ quartile | <5.2 | | 117 | 91 | 1.00 |  |
| 2^nd^ quartile | ≥5.2-<20 | | 109 | 100 | 0.70 | 0.45-1.07 |
| 3^rd^ quartile | ≥20-<46 | | 118 | 91 | 0.78 | 0.51-1.19 |
| 4^th^ quartile | ≥46 | | 123 | 85 | 0.73 | 0.47-1.15 |
|  | | | | | | |
| 5-year latency | Continuous (log Intensity rating years) | | 467 | 367 | 0.96 | 0.87-1.07 |
| 1^st^ quartile | <4.1 | | 115 | 93 | 1.00 |  |
| 2^nd^ quartile | ≥4.1-<18.6 | | 113 | 96 | 0.83 | 0.55-1.28 |
| 3^rd^ quartile | ≥18.6-<44.7 | | 116 | 93 | 0.79 | 0.51-1.22 |
| 4^th^ quartile | ≥44.7 | | 123 | 85 | 0.80 | 0.51-1.26 |
|  | | | | | | |
| 10-year latency | Continuous (log Intensity rating years) | | 467 | 367 | 0.97 | 0.87-1.08 |
| 1^st^ quartile | <2.4 | | 119 | 89 | 1.00 |  |
| 2^nd^ quartile | ≥2.4-<14.4 | | 104 | 105 | **0.64** | **0.42-0.98** |
| 3^rd^ quartile | ≥14.4-<40.8 | | 118 | 91 | 0.83 | 0.53-1.30 |
| 4^th^ quartile | ≥40.8 | | 126 | 82 | 0.83 | 0.51-1.36 |
|  | | | | | | |
| 1-4-year time window | Continuous (log Intensity rating years) | | 467 | 367 | 0.85 | 0.72-1.00 |
| <75^th^ percentile | <2.2 | | 358 | 268 | 1.00 |  |
| >75^th^ – 90^th^ percentile | ≥2.2-<6.2 | | 65 | 60 | 0.83 | 0.55-1.26 |
| >90^th^ percentile | ≥6.2 | | 44 | 39 | 0.74 | 0.46-1.19 |
|  | | | | | | |
| 5-9-year time window | Continuous (log Intensity rating years) | | 467 | 367 | 0.88 | 0.75-1.03 |
| <75^th^ percentile | <2.1 | | 359 | 267 | 1.00 |  |
| >75^th^ – 90^th^ percentile | ≥2.1-<7.8 | | 63 | 62 | 0.67 | 0.44-1.02 |
| >90^th^ percentile | ≥7.8 | | 45 | 38 | 0.77 | 0.47-1.28 |
|  | | | | | | |
| Glioma Grade 1^+^ | Continuous (log Intensity rating years) | | 4 |  | 0.65 | 0.25-1.69 |
| Glioma Grade 2^+^ | Continuous (log Intensity rating years) | | 75 |  | 0.98 | 0.80-1.20 |
| Glioma Grade 3^+^ | Continuous (log Intensity rating years) | | 67 |  | 0.94 | 0.76-1.15 |
| Glioma Grade 4^+^ | Continuous (log Intensity rating years) | | 303 |  | 0.98 | 0.87-1.10 |

^*^Assessed intensity rating × Years of exposure

†Adjusted for relatedness between cases and controls, sex, age, ethnicity, education level, smoking status, and alcohol consumption

^+^ Glioma grade adjusted for sex, age and smoking status

Table S2: Risk of glioma relative to exposure to RF EMF based on exposure estimates from the INTEROCC JEM (magnetic field)

|  | | RF EMF exposure (Intensity rating years) | Cases | Controls | OR† | 95% CI |
| --- | --- | --- | --- | --- | --- | --- |
| INTEROCC job exposure matrix (magnetic field)* | | | | | | |
| Lifetime | Continuous (log Intensity rating years) | | 467 | 367 | 1.03 | 0.91-1.16 |
| 1^st^ quartile | <0.8 | | 121 | 87 | 1.00 |  |
| 2^nd^ quartile | ≥0.8-<2.4 | | 103 | 106 | 0.67 | 0.45-1.04 |
| 3^rd^ quartile | ≥2.4-<7.1 | | 115 | 94 | 0.86 | 0.56-1.32 |
| 4^th^ quartile | ≥7.1 | | 128 | 80 | 0.92 | 0.58-1.45 |
|  | | | | | | |
| 2-year latency | Continuous (log Intensity rating years) | | 467 | 367 | 1.02 | 0.91-1.15 |
| 1^st^ quartile | <0.7 | | 119 | 89 | 1.00 |  |
| 2^nd^ quartile | ≥0.7-<2.3 | | 103 | 106 | **0.64** | **0.42-0.97** |
| 3^rd^ quartile | ≥2.3-<6.9 | | 117 | 92 | 0.87 | 0.56-1.33 |
| 4^th^ quartile | ≥6.9 | | 128 | 80 | 0.89 | 0.57-1.41 |
|  | | | | | | |
| 5-year latency | Continuous (log Intensity rating years) | | 467 | 367 | 1.03 | 0.92-1.16 |
| 1^st^ quartile | <0.7 | | 117 | 91 | 1.00 |  |
| 2^nd^ quartile | ≥0.7-<2.2 | | 106 | 103 | 0.76 | 0.5-1.16 |
| 3^rd^ quartile | ≥2.2-<6.8 | | 114 | 95 | 0.87 | 0.56-1.34 |
| 4^th^ quartile | ≥6.8 | | 130 | 78 | 1.03 | 0.65-1.64 |
|  | | | | | | |
| 10-year latency | Continuous (log Intensity rating years) | | 467 | 367 | 1.05 | 0.93-1.18 |
| 1^st^ quartile | <0.49 | | 117 | 91 | 1.00 |  |
| 2^nd^ quartile | ≥0.49-<1.9 | | 108 | 101 | 0.83 | 0.54-1.27 |
| 3^rd^ quartile | ≥1.9-<5.9 | | 114 | 95 | 0.89 | 0.57-1.38 |
| 4^th^ quartile | ≥5.9 | | 128 | 80 | 0.98 | 0.61-1.58 |
|  | | | | | | |
| 1-4-year time window | Continuous (log Intensity rating years) | | 467 | 367 | 0.85 | 0.66-1.11 |
| <75^th^ percentile | <0.2 | | 362 | 264 | 1.00 |  |
| >75^th^ – 90^th^ percentile | ≥0.2-<0.8 | | 60 | 65 | 0.68 | 0.46-1.01 |
| >90^th^ percentile | ≥0.8 | | 45 | 38 | 0.85 | 0.51-1.42 |
|  | | | | | | |
| 5-9-year time window | Continuous (log Intensity rating years) | | 467 | 367 | 0.98 | 0.74-1.31 |
| <75^th^ percentile | <0.2 | | 370 | 274 | 1.00 |  |
| >75^th^ – 90^th^ percentile | ≥0.2-<0.95 | | 56 | 59 | **0.53** | **0.34-0.84** |
| >90^th^ percentile | ≥0.95 | | 41 | 34 | 0.92 | 0.54-1.56 |
|  | | | | | | |
| Glioma Grade 1^+^ | Continuous (log Intensity rating years) | | 4 |  | 1.24 | 0.35-4.44 |
| Glioma Grade 2^+^ | Continuous (log Intensity rating years) | | 75 |  | 1.05 | 0.83-1.34 |
| Glioma Grade 3^+^ | Continuous (log Intensity rating years) | | 67 |  | 0.85 | 0.66-1.11 |
| Glioma Grade 4^+^ | Continuous (log Intensity rating years) | | 303 |  | 1.05 | 0.93-1.19 |

^*^Assessed intensity rating × Years of exposure
†Adjusted for relatedness between cases and controls, sex, age, ethnicity, education level, smoking status and alcohol consumption ^+^ Glioma grade adjusted for sex, age and smoking status

Table S3: Risk of glioma relative to exposure to RF EMF based on exposure estimates from CANJEM

|  | | RF EMF exposure (Intensity rating years) | Cases | Controls | OR† | 95% CI |
| --- | --- | --- | --- | --- | --- | --- |
| Canadian Job exposure matrix* | | | | | | |
| Lifetime | Continuous (log Intensity rating years) | | 467 | 367 | 0.98 | 0.93-1.03 |
| 1^st^ quartile | <0.081 | | 127 | 81 | 1.00 |  |
| 2^nd^ quartile | ≥0.081-<1.26 | | 115 | 94 | 0.75 | 0.50-1.14 |
| 3^rd^ quartile | ≥1.26-<12.80 | | 109 | 100 | 0.81 | 0.53-1.25 |
| 4^th^ quartile | ≥12.80 | | 116 | 92 | 0.85 | 0.54-1.32 |
|  | | | | | | |
| 2-year latency | Continuous (log Intensity rating years) | | 467 | 367 | 0.99 | 0.93-1.04 |
| 1^st^ quartile | <0.059 | | 121 | 87 | 1.00 |  |
| 2^nd^ quartile | ≥0.059-<1.12 | | 117 | 92 | 0.80 | 0.53-1.22 |
| 3^rd^ quartile | ≥1.12-<12.20 | | 114 | 95 | 0.92 | 0.60-1.41 |
| 4^th^ quartile | ≥12.20 | | 115 | 93 | 0.86 | 0.56-1.34 |
|  | | | | | | |
| 5-year latency | Continuous (log Intensity rating years) | | 467 | 367 | 0.98 | 0.95-1.02 |
| 1^st^ quartile | <0.016 | | 122 | 89 | 1.00 |  |
| 2^nd^ quartile | ≥0.016-<0.93 | | 115 | 94 | 0.74 | 0.48-1.13 |
| 3^rd^ quartile | ≥0.93-<12.20 | | 117 | 92 | 0.89 | 0.58-1.36 |
| 4^th^ quartile | ≥12.20 | | 113 | 95 | 0.85 | 0.55-1.34 |
|  | | | | | | |
| 10-year latency | Continuous (log Intensity rating years) | | 467 | 367 | 1.00 | 0.95-1.05 |
| 1^st^ quartile | <0.022 | | 143 | 102 | 1.00 |  |
| 2^nd^ quartile | ≥0.022-<0.60 | | 95 | 77 | 0.86 | 0.56-1.34 |
| 3^rd^ quartile | ≥0.60-<8.36 | | 118 | 91 | 0.98 | 0.66-1.45 |
| 4^th^ quartile | ≥8.36 | | 111 | 97 | 0.84 | 0.55-1.28 |
|  | | | | | | |
| 1-4-year time window | Continuous (log Intensity rating years) | | 467 | 367 | 0.95 | 0.89-1.01 |
| <75^th^ percentile | <0.025 | | 361 | 265 | 1.00 |  |
| >75^th^ – 90^th^ percentile | ≥0.025-<3.37 | | 71 | 54 | 0.83 | 0.54-1.26 |
| >90^th^ percentile | ≥3.37 | | 35 | 48 | 0.63 | 0.38-1.03 |
|  | | | | | | |
| 5-9-year time window | Continuous (log Intensity rating years) | | 467 | 367 | 0.97 | 0.91-1.05 |
| <75^th^ percentile | <0.031 | | 348 | 278 | 1.00 |  |
| >75^th^ – 90^th^ percentile | ≥0.031-<1.09 | | 76 | 49 | 0.95 | 0.62-1.46 |
| >90^th^ percentile | ≥1.09 | | 43 | 40 | 0.85 | 0.51-1.42 |
|  | | | | | | |
| Glioma Grade 1^+^ | Continuous (log Intensity rating years) | | 4 |  | 1.03 | 0.61-1.72 |
| Glioma Grade 2^+^ | Continuous (log Intensity rating years) | | 75 |  | 0.95 | 0.87-1.05 |
| Glioma Grade 3^+^ | Continuous (log Intensity rating years) | | 67 |  | 1.03 | 0.93-1.13 |
| Glioma Grade 4^+^ | Continuous (log Intensity rating years) | | 303 |  | 0.96 | 0.91-1.01 |

^*^Exposure prevalence x Assessed intensity rating × Years of exposure

†Adjusted for relatedness between cases and controls, sex, age, ethnicity, education level, smoking status and alcohol consumption

^+^Glioma grade adjusted for sex, age and smoking status

**Sensitivity analysis**

The data were also tested for the impact of missing data for age, alcohol use, and education. For age variable, missing data was estimated for 6 participants based on their age at the time of their last job plus the years they worked in that job (noting that the age variable was missing in demographic details, however, age was reported at the commencement of each job). Missing data for alcohol and education were added based on the median number for these variables. For alcohol missing data were replaced for 22 participants, and 28 participants for education. A sensitivity analysis was also done to determine the impact that related controls had on the outcome of the logistic regression model. This was tested by doing an analysis that only included unrelated controls (removal of 228 genetically related controls).

Table S4: Sensitivity analysis for missing data

|  | | RF EMF exposure (Intensity rating years) | Cases | Controls | OR† | 95% CI |
| --- | --- | --- | --- | --- | --- | --- |
| INTEROCC job exposure matrix (electric field)* | | | | | | |
| Lifetime | Continuous (log Intensity rating years) | | 467 | 367 | 0.96 | 0.86-1.07 |
| 2-year latency | Continuous (log Intensity rating years) | | 467 | 367 | 0.97 | 0.87-1.08 |
| 5-year latency | Continuous (log Intensity rating years) | | 467 | 367 | 0.96 | 0.86-1.07 |
| 10-year latency | Continuous (log Intensity rating years) | | 467 | 367 | 0.97 | 0.87-1.08 |
| 1-4-year time window | Continuous (log Intensity rating years) | | 467 | 367 | **0.83** | **0.70-0.97** |
| 5-9-year time window | Continuous (log Intensity rating years) | | 467 | 367 | 0.87 | 0.75-1.02 |
| INTEROCC job exposure matrix (magnetic field)* | | | | | | |
| Lifetime | Continuous (log Intensity rating years) | | 467 | 367 | 1.03 | 0.92-1.15 |
| 2-year latency | Continuous (log Intensity rating years) | | 467 | 367 | 1.02 | 0.91-1.15 |
| 5-year latency | Continuous (log Intensity rating years) | | 467 | 367 | 1.03 | 0.92-1.15 |
| 10-year latency | Continuous (log Intensity rating years) | | 467 | 367 | 1.04 | 0.93-1.17 |
| 1-4-year time window | Continuous (log Intensity rating years) | | 467 | 367 | 0.84 | 0.65-1.09 |
| 5-9-year time window | Continuous (log Intensity rating years) | | 467 | 367 | 0.97 | 0.74-1.27 |
| CANJEM^+^ | | | | | | |
| Lifetime | Continuous (log Intensity rating years) | | 467 | 367 | 0.98 | 0.94-1.04 |
| 2-year latency | Continuous (log Intensity rating years) | | 467 | 367 | 0.99 | 0.94-1.04 |
| 5-year latency | Continuous (log Intensity rating years) | | 467 | 367 | 0.98 | 0.93-1.03 |
| 10-year latency | Continuous (log Intensity rating years) | | 467 | 367 | 1.00 | 0.95-1.05 |
| 1-4-year time window | Continuous (log Intensity rating years) | | 467 | 367 | 0.95 | 0.89-1.01 |
| 5-9-year time window | Continuous (log Intensity rating years) | | 467 | 367 | 0.97 | 0.91-1.04 |

^*^ Assessed intensity rating × Years of exposure
^+^ Exposure prevalence x Assessed intensity rating × Years of exposure

†Adjusted for relatedness between cases and controls, sex, age, ethnicity, education level, smoking status and alcohol consumption

Table S5: Sensitivity analyses for unrelated controls INTEROCC JEM (electric field)

|  | | RF EMF exposure (Intensity rating years) | Cases | Controls | OR† | 95% CI |
| --- | --- | --- | --- | --- | --- | --- |
| INTEROCC job exposure matrix (electric field)* | | | | | | |
| Lifetime | Continuous (log Intensity rating years) | | 467 | 146 | 0.95 | 0.79-1.13 |
| 1^st^ quartile | >5.8 | | 120 | 33 | 1.00 |  |
| 2^nd^ quartile | ≥5.8-<21 | | 111 | 42 | **0.36** | **0.16-0.79** |
| 3^rd^ quartile | ≥21-<47.9 | | 118 | 36 | 0.61 | 0.26-1.41 |
| 4^th^ quartile | ≥47.9 | | 118 | 35 | 0.46 | 0.20-1.07 |
|  | | | | | | |
| 2-year latency | Continuous (log Intensity rating years) | | 467 | 146 | 0.94 | 0.79-1.13 |
| 1^st^ quartile | >5.3 | | 119 | 34 | 1.00 |  |
| 2^nd^ quartile | ≥5.3-<20.9 | | 112 | 42 | **0.38** | **0.17-0.84** |
| 3^rd^ quartile | ≥20.9-<46.9 | | 118 | 35 | 0.63 | 0.27-1.47 |
| 4^th^ quartile | ≥40.9 | | 118 | 35 | 0.48 | 0.21-1.11 |
|  | | | | | | |
| 5-year latency | Continuous (log Intensity rating years) | | 467 | 146 | 0.92 | 0.78-1.10 |
| 1^st^ quartile | <4.5 | | 118 | 35 | 1.00 |  |
| 2^nd^ quartile | ≥4.5-<20 | | 113 | 40 | 0.50 | 0.23-1.06 |
| 3^rd^ quartile | ≥20-<46 | | 123 | 38 | 0.74 | 0.32-1.68 |
| 4^th^ quartile | ≥46 | | 113 | 33 | 0.53 | 0.23-1.20 |
|  | | | | | | |
| 10-year latency | Continuous (log Intensity rating years) | | 467 | 146 | 0.96 | 0.82-1.14 |
| 1^st^ quartile | <2.5 | | 118 | 33 | 1.00 |  |
| 2^nd^ quartile | ≥2.5-<16.4 | | 113 | 40 | **0.31** | **0.14-0.66** |
| 3^rd^ quartile | ≥16.4-<43 | | 116 | 38 | 0.68 | 0.28-1.64 |
| 4^th^ quartile | ≥43 | | 118 | 35 | 0.45 | 0.20-1.05 |
|  | | | | | | |
| 1-4-year time window | Continuous (log Intensity rating years) | | 467 | 146 | 1.07 | 0.82-1.39 |
| <75^th^ percentile | <2.2 | | 355 | 105 | 1.00 |  |
| >75^th^ – 90^th^ percentile | ≥2.2-<6 | | 66 | 26 | 1.19 | 0.61-2.35 |
| >90^th^ percentile | ≥6 | | 46 | 15 | 1.48 | 0.64-2.35 |
|  |  | |  |  |  |  |
| 5-9-year time window | Continuous (log Intensity rating years) | | 467 | 146 | 0.90 | 0.70-1.15 |
| <75^th^ percentile | <1.9 | | 355 | 107 | 1.00 |  |
| >75^th^ – 90^th^ percentile | ≥1.9-<7.7 | | 66 | 23 | 0.54 | 0.26-1.14 |
| >90^th^ percentile | ≥7.7 | | 46 | 16 | 0.76 | 0.35-1.64 |

^*^Assessed intensity rating × Years of exposure
^+^ Exposure prevalence x Assessed intensity rating × Years of exposure

†Adjusted for relatedness between cases and controls, sex, age, ethnicity, education level, smoking status, and alcohol consumption

Table S6: Sensitivity analyses for unrelated controls INTEROCC JEM (magnetic field)

|  | | RF EMF exposure (Intensity rating years) | Cases | Controls | OR† | 95% CI |
| --- | --- | --- | --- | --- | --- | --- |
| INTEROCC job exposure matrix (magnetic field)* | | | | | | |
| Lifetime | Continuous (log Intensity rating years) | | 467 | 146 | 1.18 | 0.96-1.44 |
| 1^st^ quartile | <0.82 | | 121 | 32 | 1.00 |  |
| 2^nd^ quartile | ≥0.82-<2.6 | | 110 | 43 | 0.69 | 0.33-1.46 |
| 3^rd^ quartile | ≥2.6-<8.5 | | 120 | 34 | 0.89 | 0.43-1.84 |
| 4^th^ quartile | ≥8.5 | | 116 | 37 | 1.34 | 0.58-3.10 |
|  | | | | | | |
| 2-year latency | Continuous (log Intensity rating years) | | 467 | 146 | 1.17 | 0.96-1.43 |
| 1^st^ quartile | <0.79 | | 121 | 33 | 1.00 |  |
| 2^nd^ quartile | ≥0.79-<2.5 | | 110 | 43 | 0.56 | 0.27-1.17 |
| 3^rd^ quartile | ≥2.5-<8.4 | | 121 | 33 | 0.89 | 0.42-1.88 |
| 4^th^ quartile | ≥8.4 | | 116 | 37 | 1.28 | 0.55-2.98 |
|  | | | | | | |
| 5-year latency | Continuous (log Intensity rating years) | | 467 | 146 | 1.17 | 0.95-1.43 |
| 1^st^ quartile | <0.75 | | 120 | 33 | 1.00 |  |
| 2^nd^ quartile | ≥0.75-<2.3 | | 110 | 43 | 0.71 | 0.33-1.50 |
| 3^rd^ quartile | ≥2.3-<6.9 | | 120 | 34 | 1.03 | 0.49-2.18 |
| 4^th^ quartile | ≥6.9 | | 117 | 36 | 1.50 | 0.64-3.52 |
|  | | | | | | |
| 10-year latency | Continuous (log Intensity rating years) | | 467 | 146 | 1.17 | 0.96-1.44 |
| 1^st^ quartile | <0.5 | | 118 | 35 | 1.00 |  |
| 2^nd^ quartile | ≥0.5-<2 | | 114 | 40 | 0.51 | 0.24-1.08 |
| 3^rd^ quartile | ≥2-<6.9 | | 118 | 35 | 0.86 | 0.39-1.87 |
| 4^th^ quartile | ≥6.9 | | 117 | 36 | 1.14 | 0.49-2.65 |
|  | | | | | | |
| 1-4-year time window | Continuous (log Intensity rating years) | | 467 | 146 | 0.95 | 0.64-1.43 |
| <75^th^ percentile | <0.19 | | 355 | 104 | 1.00 |  |
| >75^th^ – 90^th^ percentile | ≥0.19-<0.76 | | 64 | 29 | 0.89 | 0.43-1.85 |
| >90^th^ percentile | ≥0.76 | | 48 | 13 | 0.79 | 0.38-1.66 |
|  | | | | | | |
| 5-9-year time window | Continuous (log Intensity rating years) | | 467 | 146 | 1.09 | 0.69-1.70 |
| <75^th^ percentile | <0.2 | | 355 | 104 | 1.00 |  |
| >75^th^ – 90^th^ percentile | ≥0.2-<0.95 | | 65 | 28 | 0.64 | 0.31-1.33 |
| >90^th^ percentile | ≥0.95 | | 47 | 14 | 0.71 | 0.31-1.61 |

^*^Assessed intensity rating × Years of exposure
^+^ Exposure prevalence x Assessed intensity rating × Years of exposure

†Adjusted for relatedness between cases and controls, sex, age, ethnicity, education level, smoking status, and alcohol consumption

Table S7: Sensitivity analyses for unrelated controls CANJEM

|  | | RF EMF exposure (Intensity rating years) | Cases | Controls | OR† | 95% CI |
| --- | --- | --- | --- | --- | --- | --- |
| CANJEM^+^ | | | | | | |
| Lifetime | Continuous (log Intensity rating years) | | 467 | 146 | 0.97 | 0.89-1.05 |
| 1^st^ quartile | <0.063 | | 123 | 30 | 1.00 |  |
| 2^nd^ quartile | ≥0.063-<1.26 | | 119 | 34 | 0.70 | 0.34-1.44 |
| 3^rd^ quartile | ≥1.26-<12.64 | | 109 | 45 | 0.70 | 0.32-1.55 |
| 4^th^ quartile | ≥12.64 | | 116 | 37 | 0.70 | 0.34-1.45 |
|  | | | | | | |
| 2-year latency | Continuous (log Intensity rating years) | | 467 | 146 | 0.97 | 0.89-1.06 |
| 1^st^ quartile | <0.059 | | 120 | 33 | 1.00 |  |
| 2^nd^ quartile | ≥0.059-<1.15 | | 122 | 32 | 0.72 | 0.35-1.49 |
| 3^rd^ quartile | ≥1.15-<12.44 | | 110 | 43 | 0.87 | 0.39-1.91 |
| 4^th^ quartile | ≥12.44 | | 115 | 38 | 0.74 | 0.36-1.52 |
|  | | | | | | |
| 5-year latency | Continuous (log Intensity rating years) | | 467 | 146 | 0.97 | 0.89-1.05 |
| 1^st^ quartile | <0.028 | | 124 | 29 | 1.00 |  |
| 2^nd^ quartile | ≥0.028-<0.97 | | 116 | 38 | 0.83 | 0.40-1.72 |
| 3^rd^ quartile | ≥0.97-<11.22 | | 114 | 39 | 0.85 | 0.40-1.82 |
| 4^th^ quartile | ≥11.22 | | 113 | 40 | 0.78 | 0.37-1.62 |
|  | | | | | | |
| 10-year latency | Continuous (log Intensity rating years) | | 467 | 146 | 0.98 | 0.90-1.06 |
| 1^st^ quartile | <0.0119 | | 145 | 33 | 1.00 |  |
| 2^nd^ quartile | ≥0.0119-<0.67 | | 96 | 33 | 1.04 | 0.50-2.13 |
| 3^rd^ quartile | ≥0.67-<8.44 | | 115 | 39 | 0.87 | 0.43-1.77 |
| 4^th^ quartile | ≥8.44 | | 111 | 41 | 0.76 | 0.38-1.53 |
|  | | | | | | |
| 1-4-year time window | Continuous (log Intensity rating years) | | 467 | 146 | 0.96 | 0.86-1.08 |
| <75^th^ percentile | <0.022 | | 357 | 103 | 1.00 |  |
| >75^th^ – 90^th^ percentile | ≥0.022-<2.37 | | 71 | 21 | 0.91 | 0.47-1.76 |
| >90^th^ percentile | ≥2.37 | | 39 | 22 | 0.77 | 0.32-1.85 |
|  | | | | | | |
| 5-9-year time window | Continuous (log Intensity rating years) | | 467 | 146 | 0.98 | 0.87-1.11 |
| <75^th^ percentile | <0.047 | | 353 | 107 | 1.00 |  |
| >75^th^ – 90^th^ percentile | ≥0.047-<0.99 | | 69 | 23 | 1.54 | 0.70-3.40 |
| >90^th^ percentile | ≥0.99 | | 45 | 16 | 0.71 | 0.34-1.52 |

^*^Assessed intensity rating × Years of exposure
^+^ Exposure prevalence x Assessed intensity rating × Years of exposure

†Adjusted for relatedness between cases and controls, sex, age, ethnicity, education level, smoking status, and alcohol consumption

Table S8: Sensitivity analyses of assigning INTEROCC JEM (E-field) exposure estimates for jobs with exposure prevalences above the median prevalence of exposure among all exposed jobs

|  | | RF EMF exposure (Intensity rating years) | Cases | Controls | OR† | 95% CI |
| --- | --- | --- | --- | --- | --- | --- |
| INTEROCC job exposure matrix (magnetic field)* | | | | | | |
| Lifetime | Continuous (log Intensity rating years) | | 467 | 367 | 0.98 | 0.88-1.10 |
| <75^th^ percentile | <0.8 | | 336 | 290 | 1.00 |  |
| >75^th^ – 90^th^ percentile | ≥0.8-<5.1 | | 76 | 49 | 0.85 | 0.55-1.31 |
| >90^th^ percentile | ≥5.1 | | 55 | 28 | 1.16 | 0.71-1.88 |
|  | | | | | | |
| 2-year latency | Continuous (log Intensity rating years) | | 467 | 367 | 1.00 | 0.98-1.03 |
| <75^th^ percentile | <0.8 | | 336 | 290 | 1.00 |  |
| >75^th^ – 90^th^ percentile | ≥0.8-<5 | | 76 | 49 | 0.81 | 0.53-1.25 |
| >90^th^ percentile | ≥5 | | 55 | 28 | 1.14 | 0.71-1.87 |
|  | | | | | | |
| 5-year latency | Continuous (log Intensity rating years) | | 467 | 367 | 1.00 | 0.97-1.03 |
| <75^th^ percentile | <0.78 | | 336 | 290 | 1.00 |  |
| >75^th^ – 90^th^ percentile | ≥0.78-<4.9 | | 78 | 47 | 0.88 | 0.57-1.35 |
| >90^th^ percentile | ≥4.9 | | 53 | 53 | 1.04 | 0.65-1.68 |
|  | | | | | | |
| 10-year latency | Continuous (log Intensity rating years) | | 467 | 367 | 1.00 | 0.97-1.03 |
| <75^th^ percentile | <0.53 | | 340 | 286 | 1.00 |  |
| >75^th^ – 90^th^ percentile | ≥0.53-<3.9 | | 76 | 49 | 0.80 | 0.52-1.22 |
| >90^th^ percentile | ≥3.9 | | 51 | 32 | 0.82 | 0.50-1.35 |

^*^ Exposure prevalence x Assessed intensity rating × Years of exposure

†Adjusted for relatedness between cases and controls, sex, age, ethnicity, education level, smoking status, and alcohol consumption

Table S9: Sensitivity analyses of assigning INTEROCC JEM (H-field) exposure estimates for jobs with exposure prevalences above the median prevalence of exposure among all exposed jobs

|  | | RF EMF exposure (Intensity rating years) | Cases | Controls | OR† | 95% CI |
| --- | --- | --- | --- | --- | --- | --- |
| INTEROCC job exposure matrix (magnetic field)* | | | | | | |
| Lifetime | Continuous (log Intensity rating years) | | 467 | 367 | 0.99 | 0.87-1.12 |
| <75^th^ percentile | <0.17 | | 335 | 291 | 1.00 |  |
| >75^th^ – 90^th^ percentile | ≥0.17-<0.87 | | 80 | 45 | 1.07 | 0.70-1.63 |
| >90^th^ percentile | ≥0.87 | | 52 | 31 | 0.84 | 0.48-1.46 |
|  | | | | | | |
| 2-year latency | Continuous (log Intensity rating years) | | 467 | 367 | 1.01 | 0.95-1.08 |
| <75^th^ percentile | <0.17 | | 334 | 292 | 1.00 |  |
| >75^th^ – 90^th^ percentile | ≥0.17-<0.85 | | 80 | 45 | 1.09 | 0.71-1.67 |
| >90^th^ percentile | ≥0.85 | | 53 | 30 | 0.87 | 0.50-1.50 |
|  | | | | | | |
| 5-year latency | Continuous (log Intensity rating years) | | 467 | 367 | 1.01 | 0.95-1.08 |
| <75^th^ percentile | <0.16 | | 331 | 295 | 1.00 |  |
| >75^th^ – 90^th^ percentile | ≥0.16-<0.83 | | 82 | 43 | 1.15 | 0.75-1.78 |
| >90^th^ percentile | ≥0.83 | | 54 | 29 | 0.95 | 0.55-1.63 |
|  | | | | | | |
| 10-year latency | Continuous (log Intensity rating years) | | 467 | 367 | 1.01 | 0.95-1.07 |
| <75^th^ percentile | <0.12 | | 328 | 298 | 1.00 |  |
| >75^th^ – 90^th^ percentile | ≥0.12-<0.76 | | 86 | 39 | 1.48 | 0.94-2.31 |
| >90^th^ percentile | ≥0.76 | | 53 | 30 | 0.95 | 0.56-1.63 |

* Exposure prevalence x Assessed intensity rating × Years of exposure

†Adjusted for relatedness between cases and controls, sex, age, ethnicity, education level, smoking status, and alcohol consumption

Table S10: Ten highest exposed occupations (intensity rating) based on the INTEROCC JEM (E-field and H-field) and CANJEM

| INTEROCC job exposure matrix (E-field) | | |
| --- | --- | --- |
| Job code and title | Intensity rating | Prevalence |
| 8269 - Other textile products machine operators | 33.97 | 0.13 |
| 4132 - Production clerks | 30.16 | 0.02 |
| 7341 - Compositors and type setters | 28.31 | 0.03 |
| 8266 - Shoemaking and related machine operators | 28.31 | 0.05 |
| 3112 - Civil engineering technicians | 20.73 | 0.03 |
| 7121 - Builders, traditional materials (new) | 13.73 | 0.04 |
| 7131 - Roofers | 13.10 | 0.04 |
| 9312 - Construction and maintenance labourers: roads, dams and similar constructions | 10.65 | 0.08 |
| 8232 -Plastic products machine operators | 10.58 | 0.03 |
| 2455 - Film, stage and related actors and directors | 10.07 | 0.12 |
| INTEROCC job exposure matrix (H-field) |  |  |
| Job code and title | Intensity rating | Prevalence |
| 8211 - Machine-tool operators | 12.69 | 0.01 |
| 8269 - Other textile products machine operators | 11.45 | 0.13 |
| 9320 - Manufacturing labourers | 10.05 | 0.03 |
| 7341 - Compositors, typesetters and related workers | 9.54 | 0.03 |
| 8266 - Shoemaking and related machine operators | 9.54 | 0.05 |
| 7212 - Welders and flame cutters | 7.80 | 0.04 |
| 8232 -Plastic products machine operators | 7.67 | 0.03 |
| 3115 - Mechanical engineering technicians | 6.95 | 0.07 |
| 9311 - Mining and quarrying labourers | 6.35 | 0.14 |
| 2221 - Medical doctors | 6.19 | 0.01 |
| CANJEM |  |  |
| Job code and title | Intensity rating | Prevalence |
| 1210 - Directors and chief executives | 25.00 | 0.17 |
| 1229 - Other specialised managers | 25.00 | 0.17 |
| 5162 - Policemen/women | 13.00 | 0.05 |
| 2332 - Pre-primary education teaching professionals | 12.71 | 0.26 |
| 3113 Electrical engineering technicians | 12.00 | 0.62 |
| 2320 - Secondary education teaching professionals | 11.05 | 0.31 |
| 3143 - Aircraft pilots and related workers | 7.00 | 0.50 |
| 3118 - Technical draughters | 6.64 | 0.65 |
| 1314 - General managers in retail and wholesale trade | 5.00 | 0.08 |
| 3114 - Electronics and telecommunications engineering technicians | 5.00 | 0.57 |
