# Supplementary material for: Is occupational exposure to radiofrequency electromagnetic fields associated with glioma risk? An Australian population-based family case–control study
Source: BMJ Open. 2026 Mar 12;16(3):e107281. doi: 10.1136/bmjopen-2025-107281 (PMC12983896; doi:10.1136/bmjopen-2025-107281)
Supplement: Supplementary file 2 [file bmjopen-2025-107281.reviewer_comments.pdf]

PEER REVIEW HISTORY

BMJ Open publishes all reviews undertaken for accepted manuscripts. Reviewers are asked to complete a checklist review form and are provided with free text boxes to elaborate on their assessment. These free text comments are reproduced below.

ARTICLE DETAILS

Title (Provisional)

Is occupational exposure to radiofrequency electromagnetic fields associated with glioma risk? An Australian population-based family case-control study

Authors

Mate, Rohan; Benke, Geza; Loughran, Sarah P; Abramson, Michael J; Vjadic, Claire; Turner, Michelle; Turuban, Maxime; Cardis, Elisabeth; Karipidis, Ken

---

VERSION 1 - REVIEW

**Reviewer**	**1**
**Name**	**Seo, Songwon**
**Affiliation**	**Korea Institute of Radiological and Medical Sciences**
**Date**	**18-Aug-2025**
**COI**	**None**

The authors conducted a case-control study assessing glioma risk in relation to occupational exposure to RF EMF, applying two job-exposure matrices (INTEROCC and CANJEM). The use of improved RF EMF exposure assessment, along with the careful consideration of latency and time windows, represents a strength of the study. However, some methodological clarifications and further discussion are required to strengthen the manuscript.

1. The manuscript appears to frame the study primarily in terms of hypothesis testing, with explicit reference to a null hypothesis and a conclusion of “no evidence.” In a case-control design, however, the emphasis is typically on estimating associations rather than formal hypothesis testing, especially in the absence of explicit statistical power considerations in the study design. It would be preferable to rephrase these sections to highlight the interpretation of odds ratios with confidence intervals (i.e., association), rather than hypothesis testing.

2. Both INTEROCC JEM and CANJEM inherently involve exposure misclassification, given their reliance mostly on job titles rather than individual-level exposure data. While the manuscript notes this as non-differential misclassification, please expand the discussion on whether the degree of misclassification might differ between the two JEMs, and how this may have influenced the comparability of their results.

3. Was the possibility of a healthy worker effect considered? Please discuss its potential impact, if any, on the findings of this study.

4. The authors assessed cumulative exposure as a continuous variable by log transformation. Please address how the zero values were handled in this transformation.

**Reviewer**	**2**
**Name**	**Modenese, Alberto**
**Affiliation**	**University of Modena and Reggio Emilia**
**Date**	**25-Aug-2025**
**COI**	**None**

I read with great interest your manuscript entitled “Occupational Exposure to Radiofrequency Electromagnetic Fields and the Risk of Glioma”.

The manuscript is well written and has high scientific value.

Please address the following required revisions before it could be considered for possible publication in BMJ Open.

Best regards,

The Reviewer

Title

Are you considering only glioma of the brain or also other locations for glioma?

Keywords

I can’t understand which are the keywords. Seem too few, and possibly not relevant.

Abstract

The results section in the abstract is too short compared to introduction and methods. Even if it is an abstract some more details are needed on how, when and where the subjects have been interviewed.

Introduction

- Page 2 Lines 43-60: in text citations here seem not following the correct order

- Page 3 line 6: glioma is the most frequent malignant brain tumor even in children, not only in adults

- Page 3 line 8: shouldn’t be “after” instead of “since”?

- Page 3 line 34: “to many more job titles” compared to what?

Methods

- Suggest to divide the methods section in sub-sections to improve readability

- Page 4 lines 31-32: more details on how and who did the ISCO-88 coding are needed here

Results

- It would be helpful to have p values in Table 1

- It seems to me that the main results are displayed in Figure 1 (and in supplementary tables), which is supposed to be the one at page 12, but I can't see any caption for the Figure. Moreover, mentioning a red color in a black and white Figure could be misleading. I understand that there is no red cause there is no significant results: any other idea to highlight the lack of significance in the Figure?

Discussion

- Please include in the Discussion a comparison with the relevant results of the recently published WHO commissioned systematic review, cited in the introduction, but not discussed here.

- Please add further limitations of the study which can have an impact on the results obtained: the case vs controls rate is > 1:1 and there is a relevant difference in sex distribution between cases and controls, with males much more represented among controls.

Conclusions

- Line 25: please add "occupational" before "RF EMF"

**Reviewer**	**3**
**Name**	**Jalilian, Hamed**
**Affiliation**	**Swiss Tropical and Public Health Institute**
**Date**	**16-Sep-2025**
**COI**	**None**

Please find my comments on the attached file.

**** The reviewer provided a marked copy with additional comments. Please contact the publisher for full details. ****

**Reviewer**	**4**
**Name**	**Leung, Isaac**
**Affiliation**	**The Chinese University of Hong Kong, Statistics**
**Date**	**03-Nov-2025**

**COI**	**None**

The case-control study studying whether Glioma is associated with occupational exposure is a challenging task. But the authors try their very best to measure the exposure and to isolate any nuisance/confounding factors. Although the result is same (no association), it gives the same result with a stronger evidence by introducing new JEMs to improve the measurement of exposure.

I have some further comments that may improve the paper:

1) May the JEMs calculations be described more clearly? (For example, which jobs or activities give which levels of exposure to the RF EMF?) (Or providing some graphs to display such a matrix?)

2) Do you think that apart from the controlled factors (i.e. sex, age, ethnicity, education level, smoking status and alcohol consumption) and jobs, are there any factors worth controlling? (for example type of living homes, leisure activities, dietary habits or some elements in their blood/bone etc?)

3) if the control is matched with the case, control and case may be similar/correlated, do you think that mixed logistic regression can account for this (nuisance) correlation?

4) In my opinion, the highly skewed distribution of the exposure of RF EMF makes statistical inference difficult (very few examples having large exposure compared with normal cases), the author has try to make things more normal by taking natural log. Is the skewness reduced much after taking natural log?

5) Can you elaborate more on how to conduct a better future study? (i.e. Can you give further details of how to "refining occupational RF EMF exposure assessment including more accurately

describing the highest exposed occupations and exposure levels and sources over time."?)

VERSION 1 - AUTHOR RESPONSE

Reviewer: 1

Use of improved RF EMF exposure assessment, along with the careful consideration of latency and time windows, represents a strength of the study. However, some methodological clarifications and further discussion are required to strengthen the manuscript.

1. *The manuscript appears to frame the study primarily in terms of hypothesis testing, with explicit reference to a null hypothesis and a conclusion of "no evidence." In a case-control design, however, the emphasis is typically on estimating associations rather than formal hypothesis testing, especially in the absence of explicit statistical power considerations in the study design. It would*

be preferable to rephrase these sections to highlight the interpretation of odds ratios with confidence intervals (i.e., association), rather than hypothesis testing.

Agreed, we have revised the text to emphasise we are estimating associations (page 5, line numbers 34-35).

2. *Both INTEROCC JEM and CANJEM inherently involve exposure misclassification, given their reliance mostly on job titles rather than individual-level exposure data. While the manuscript notes this as non-differential misclassification, please expand the discussion on whether the degree of misclassification might differ between the two JEMs, and how this may have influenced the comparability of their results.*

Agreed, further text has been added in the Discussion on the impact of misclassification between the two JEMs (page 10, line numbers 57-59).

3. *Was the possibility of a healthy worker effect considered? Please discuss its potential impact, if any, on the findings of this study.*

The cases and controls in our study were selected from the general population, not an occupational cohort, so the Healthy Worker Effect was not relevant in our study.

4. *The authors assessed cumulative exposure as a continuous variable by log transformation. Please address how the zero values were handled in this transformation.*

We added an offset of plus 1 to all values. We have now added text in the Methods to make this clear (page 7, line number 14).

Reviewer: 2

I read with great interest your manuscript entitled "Occupational Exposure to Radiofrequency Electromagnetic Fields and the Risk of Glioma". The manuscript is well written and has high scientific value. Please address the following required revisions before it could be considered for possible publication in BMJ Open.

5. *Title: Are you considering only glioma of the brain or also other locations for glioma?*

We are only considering glioma of the brain.

6. *Keywords: I can't understand which are the keywords. Seem too few, and possibly not relevant.*

Agreed, the keywords have been revised (they were chosen from a drop-down list in the online submission portal). The Key words are: radiofrequency electromagnetic fields, occupational exposure, job-exposure matrix, brain cancer.

7. *Abstract: The results section in the abstract is too short compared to introduction and methods. Even if it is an abstract some more details are needed on how, when and where the subjects have been interviewed.*

Agreed, we have provided more detail in the Abstract as requested (page 2, line numbers 38-60).

Introduction:

8. *- Page 2 Lines 43-60: in text citations here seem not following the correct order*

Agreed, citation order revised.

9. *Page 3 line 6: glioma is the most frequent malignant brain tumor even in children, not only in adults*

Agreed, we have removed "adults" so it indicates that it is the most frequent malignant brain tumour regardless of age group (page 4, line number 51).

10. *Page 3 line 8: shouldn't be "after" instead of "since"?*

Thank you. We have changed the text to "Subsequent to the WHO systematic review, .." (page 4, line number 55).

11. *Page 3 line 34: "to many more job titles" compared to what?*

Agreed, we have revised the sentence to indicate "more job titles compared to previous JEMs" (page 5, line number 25).

Methods

12. *Suggest to divide the methods section in sub-sections to improve readability*

Agreed, sub-sections have now been included as suggested.

13. *Page 4 lines 31-32: more details on how and who did the ISCO-88 coding are needed here*

Agreed, revised as suggested (page 6, line numbers 35-36).

Results

14. *It would be helpful to have p values in Table 1*

Agreed, p-values are now included in Table 1 (page 8, line numbers 10-38).

15. *It seems to me that the main results are displayed in Figure 1 (and in supplementary tables), which is supposed to be the one at page 12, but I can't see any caption for the Figure. Moreover, mentioning a red color in a black and white Figure could be misleading. I understand that there is no red cause there is no significant results: any other idea to highlight the lack of significance in the Figure?*

We included a caption, but for some reason it wasn't included in the Proof PDF and we didn't notice. We will make sure this does not happen in the revised draft and that the caption is shown. The Figure included three significant results in red, but we understand that these might be difficult to see, and we replaced the red dots with crosses.

Discussion

16. *Please include in the Discussion a comparison with the relevant results of the recently published WHO commissioned systematic review, cited in the introduction, but not discussed here.*

A comparison with the studies included in the WHO commissioned systematic review was made in the second paragraph of the Discussion. However, we have now revised the text to make this clearer (page number 9, line numbers 35-50).

17. *Please add further limitations of the study which can have an impact on the results obtained: the case vs controls rate is > 1:1 and there is a relevant difference in sex distribution between cases and controls, with males much more represented among controls.*

Agreed, text has been added to the Discussion addressing the case/control imbalance as well as the gender imbalance between cases and controls (page 10, line numbers 46-55).

Conclusions

18. - *Line 25: please add "occupational" before "RF EMF"*

Agreed, revised as suggested (page 11, line number 33).

Reviewer: 3

Please find my comments on the attached file.

1. *Page 2, line 4, maybe if you change the Glioma to Brain tumor, make the paper more visible latter*

Our study did not investigate other types of brain tumour (such as meningiomas, acoustic neuromas etc). We have not made any changes in response to this comment as it is important to mention the precise disease investigated.

2. *Page 2, line 7, should not be 1, 2?*

Agreed, references have been revised (page 4, line number 21-27).

3. *Page 2, line 44, KHz*

The correct abbreviation for "kilo" is "k". See https://www.nist.gov/pml/owm/metric-si-prefixes

4. *Page 2, line 52, report typical exposures in diffident jobs with high rf emf exposure*

Agreed, we have added typical high RF EMF exposures (page 4, line numbers 32-38).

5. *Page 2, line 57, this is not ICNIRP reference*

Agreed, the reference has been corrected.

6. *Page 3, line 8, While the recent WHO study showed no association, you need better justification for your study and just examin Turuban study is not enough*

Agreed, the WHO systematic review included only 3 studies, so there was really limited evidence on this topic. We have now added text to indicate the limited evidence as a justification (page 4, line numbers 54-55).

7. *Page 3, line 20, Reference*

Agreed, reference added (page 4, line number 38).

8. *Page 3, line 24, I agree with the statement but you also going to use JEM that is a title-based tool. what is the diffidence of your method with the current literature method?*

We agree the JEMs rely on job titles in order to apply exposure estimates and this is prone to non-differential misclassification. This limitation is mentioned in the Discussion (page 11, line numbers 3-12). The advantage of the JEMs we used is they classify many occupations compared to previous methods and this is mentioned in the Introduction (page 5, line numbers 8-28).

9. *Page 4, Lines 42-46, I think this is not a correct approach to categorize the exposure. this method will end up with exposure misclassification. you calculate cumulative exposure by adding years of exposure, without considering the LEVEL of exposure. it means that a shop keeper as a exposed person (you mentioned above) might be in a Q4 but a welder (that is a high exposed job) might be in Q1, according to years of exposure. So I think you need to relanalyz your data becuse the results are based on this approach. also I could not see any information about exposure level in different jobs like low, medium and high RF electric or magnetic fields exposed job.*

The cumulative exposure in our study was calculated by multiplying the intensity rating of exposure with the duration of exposure (Intensity rating x Duration = Cumulative exposure). Because the intensity rating of exposure in both the INTEROCC JEM and CANJEM was unitless, the unit for the resulting cumulative exposure was "years". However, we understand that this can be confusing so we have changed the units of the cumulative exposure to "intensity rating years". Further we have added a formula in the text on how the cumulative exposure was calculated to make it clear (line number 160).

We were supplied the JEMs to use in our study, but the underlying algorithms are not open access. Nevertheless, we have now included a table in the Appendix that provides the JEM exposure estimates of the 10 highest exposed occupations using each JEM (Table S10).

10. *Page 5, line 10, add you key figures in the main text. all figures and tables of findings are in the appendix*

The figure with all the results (Figure 1) is in the main text and only more detailed results are in the Appendix.

11. *Page 5, line 31, you only need a column for cases include the number of cases and in parenthesis percentage, and a column for control. you also might consider statistical tests to show the signification of diffidence between case and controls for charismatics.*

Agreed, revised as suggested (page 8, line numbers 8-38).

12. *Page 6, line 3, in th results section I expceted to see RF EMF (100 kHz to 300 GHz) exposed populations. Becuse in the paper you only disccussed this range of frequency. But in the result you presented electric and magnetic fields that is under 100 kHz.*

We only used the RF component of INTEROCC, i.e. E-field and H-field estimates in the frequency range 100 kHz to 300 GHz

13. *Page 6, line 15, we have magnetic field almost anywhere we have electric field. so how you can explain this difference 20 vs 3 years?*

The INTEROCC JEM intensity ratings are calculated as squared ratios of the time weighted average (TWA) exposure level and the ICNIRP reference levels. Because of this, INTEROCC provides different intensity ratings for the E-field and H-field components for different occupations. Exposures to TWA E-field tend to be much higher in relation to the ICNIRP E-field reference level compared to TWA H-field exposures in relation to the ICNIRP H-field reference level. For example, the highest intensity rating in the E-field component of INTEROCC is 34 (for other textile machine operators) compared to 13 for the H-field component (for machine-tool operators). We have added text in the Methods and Discussion to explain the disparity in the median cumulative exposure when applying the E-field and H-field components of INTEROCC (page 6, line numbers 16-19 and page 10, line numbers 19-23).

14. *Page 6, line 16, electric or magnetic field or RF EMF?*

CANJEM only provides exposure estimates to RF EMF and not separate E-field and H-field components. The text on the explanation of CANJEM in the Methods has been revised to make this clear (page 6, line numbers 31-33).

15. *Page 6, line 53, any literature finding on this? any justification for these findings?*

Agreed, we have provided a reference on the known biomechanisms of RF EMF (page line number 21).

We already describe in the text that given the multiple comparisons conducted in this study (66 of them), we could not exclude that the inverse associations may have arisen by chance (page 9, line numbers 33-40).

16. *Page 6, line 56, just discuss it without saying for example*

Agreed, revised as suggested (page 9, line number 42).

17. *Page 13, line 28, if it is electric field, only use electric field not RF EMF then mention electric field in parentheses. apply this for all pictures. also in all relevant text in whole manuscript. Distinguish RF EMF, electric field, magnetic field. It is very difficult to follow them.*

Agreed, revised as suggested (revised throughout).

Reviewer: 4

The case-control study studying whether Glioma is associated with occupational exposure is a challenging task. But the authors try their very best to measure the exposure and to isolate any nuisance/confounding factors. Although the result is same (no association), it gives the same result with a stronger evidence by introducing new JEMs to improve the measurement of exposure.

1) May the JEMs calculations be described more clearly? (For example, which jobs or activities give which levels of exposure to the RF EMF?) (Or providing some graphs to display such a matrix?)

Agreed, we have added further text describing the JEMs as well as the calculation of cumulative exposure (page 6, line numbers 35-56).

The INTEROCC JEM and CANJEM were developed by other research groups. Although we were supplied with the JEMs to use in our study, the information in them is not open access. Nevertheless, we have now included a table in the Appendix that provides the estimates of the 10 highest exposed occupations of the two JEMs

2) Do you think that apart from the controlled factors (i.e. sex, age, ethnicity, education level, smoking status and alcohol consumption) and jobs, are there any factors worth controlling? (for example type of living homes, leisure activities, dietary habits or some elements in their blood/bone etc?)

We controlled for study design and confounding factors collected from study participants. We do not have data on home construction, leisure activities, dietary habits or blood/bone markers. In the Discussion we point out the possibility of uncontrolled confounding (page 11, line numbers 14-29).

> *3) if the control is matched with the case, control and case may be similar/correlated, do you think that mixed logistic regression can account for this (nuisance) correlation?*

We agree that in our study cases and controls may be similar/correlated because the cases are partners/siblings of the cases. That is why we applied the variance-covariance matrix of the estimators (VCE) cluster option in the model to account for potential correlations between cases related to controls. The VCE cluster option in family case–control studies is applied to correct the standard errors for within-family correlation, ensuring valid inference (see also response to comment from the editor) (page 7, line numbers 21-23).

> *4) In my opinion, the highly skewed distribution of the exposure of RF EMF makes statistical inference difficult (very few examples having large exposure compared with normal cases), the author has try to make things more normal by taking natural log. Is the skewness reduced much after taking natural log?*

Log-transforming reduced the skewness, especially for the INTEROCC JEM E-field data, but less so for the INTEROCC H-field and CANJEM.

> *5) Can you elaborate more on how to conduct a better future study? (i.e. Can you give further details of how to "refining occupational RF EMF exposure assessment including more accurately describing the highest exposed occupations and exposure levels and sources over time."?)*

Agreed, further text has been added to elaborate on future research (page 11, line numbers 37-40).

VERSION 2 - REVIEW

Reviewer 1

Name Seo, Songwon

Affiliation Korea Institute of Radiological and Medical Sciences

Date 29-Dec-2025

COI

The revised manuscript is clearer and more transparent, and the authors have addressed the methodological limitations raised previously. However, several of the methodological issues originally raised have been addressed in a somewhat simplified manner. Further clarification, potentially supported by additional analyses where feasible, would strengthen the interpretation of the findings.

1. Both INTEROCC and CANJEM rely primarily on job titles rather than individual-level exposure data and therefore inherently involve exposure misclassification. The revised Discussion appropriately acknowledges this limitation and notes that such misclassification is likely non-differential. However, my original concern was not only the presence of misclassification, but whether the degree or nature of misclassification may differ between the two JEMs, given differences in how exposures are derived, assigned, and applied across occupational categories. While these structural differences are now described more clearly, it remains unclear how they may have influenced the comparability of results derived from the two JEMs. Further discussion of how differences in exposure prevalence, exposure contrast, or intensity assignment could have biased risk estimates in similar or different directions would strengthen the interpretation. In addition, the application of the INTEROCC JEM resulted in a very high prevalence of classified RF EMF exposure. Although sensitivity analyses restricted to higher-exposure occupations were conducted, the implications of limited exposure contrast for the ability to detect associations are not fully explored. This issue is central to the interpretation of largely null findings and would benefit from a clearer explanation in the Discussion.

2. The authors state that the healthy worker effect is not relevant because the study is population-based rather than an occupational cohort. While this argument is reasonable with respect to the classical healthy worker effect, it may oversimplify the issue in the context of occupational exposure. Even in population-based case–control studies, cumulative exposure metrics and long-term employment in higher-exposure occupations may still be influenced by healthy worker survivor effects. Distinguishing between these concepts and addressing their potential implications for exposure patterns and risk estimates would improve the discussion of limitations and the interpretation of the study findings

3. The authors indicate that cumulative exposure was log-transformed after adding a constant of one to zero values. While this approach is commonly used, the choice of the constant may influence the transformed exposure distribution, particularly at lower exposure levels, given that the cumulative exposure metric is unitless. Providing a

justification for this choice would strengthen methodological transparency and robustness of the study findings.

Reviewer 3
Name Jalilian, Hamed
Affiliation Swiss Tropical and Public Health Institute
Date 12-Jan-2026
COI

None

Reviewer 4
Name Leung, Isaac
Affiliation The Chinese University of Hong Kong, Statistics
Date 04-Jan-2026
COI

The author has adequately addressed all my questions, include

1) Showing the detailed steps and formulae to calculate cumulative exposure (with details and support to the intensity ratings in supplementary materials)

2) Through VCE, taking into account the correlation within family cluster when doing logistic regression.

3) Adequate contents in limitations and discussion.

VERSION 2 - AUTHOR RESPONSE

Reviewer: 1
Comments to the Author:
The revised manuscript is clearer and more transparent, and the authors have addressed the methodological limitations raised previously. However, several of the methodological issues originally raised have been addressed in a somewhat simplified manner. Further clarification, potentially supported by additional analyses where feasible, would strengthen the interpretation of the findings.
1. Both INTEROCC and CANJEM rely primarily on job titles rather than individual-level exposure data and therefore inherently involve exposure misclassification. The revised Discussion appropriately acknowledges this limitation and notes that such misclassification is likely non-differential. However, my original concern was not only the presence of misclassification, but whether the degree or nature of misclassification may differ between the two JEMs, given differences in how exposures are derived, assigned, and applied across occupational categories. While these structural differences are now described more clearly, it remains unclear how they may have influenced the comparability of results derived from the two JEMs. Further discussion of how differences in exposure prevalence, exposure contrast, or intensity assignment could have biased risk estimates in similar or different directions would strengthen the interpretation. In addition, the application of the INTEROCC JEM resulted in a very high prevalence of classified RF EMF exposure. Although sensitivity analyses restricted to higher-exposure

occupations were conducted, the implications of limited exposure contrast for the ability to detect associations are not fully explored. This issue is central to the interpretation of largely null findings and would benefit from a clearer explanation in the Discussion.

Agreed. We have added text to indicate the impact that the differences in exposure prevalence, intensity and contrast made on the degree of misclassification between the two JEMs and the results overall (page 9, lines 3-14).

2. The authors state that the healthy worker effect is not relevant because the study is population-based rather than an occupational cohort. While this argument is reasonable with respect to the classical healthy worker effect, it may oversimplify the issue in the context of occupational exposure. Even in population-based case–control studies, cumulative exposure metrics and long-term employment in higher-exposure occupations may still be influenced by healthy worker survivor effects. Distinguishing between these concepts and addressing their potential implications for exposure patterns and risk estimates would improve the discussion of limitations and the interpretation of the study findings

Agreed. We have clarified the potential for the healthy worker effect in the revised manuscript (page 8, lines 33-40). We argue this bias was reduced in our study because: (a) The use of family controls ensured cases and controls shared similar lifestyle and socioeconomic backgrounds and health-seeking behaviours, providing a more comparable baseline than general population controls , and (b) the rapid clinical onset of glioma suggested that cases were likely active in the workforce until shortly before diagnosis, reducing selection bias related to health-related unemployment.

3. The authors indicate that cumulative exposure was log-transformed after adding a constant of one to zero values. While this approach is commonly used, the choice of the constant may influence the transformed exposure distribution, particularly at lower exposure levels, given that the cumulative exposure metric is unitless. Providing a justification for this choice would strengthen methodological transparency and robustness of the study findings.

Agreed. We now clarify that adding 1 was the minimum offset that could be used in order to preserve exposure values ≥ 0 whilst keeping distortion of the distribution at lower levels at a minimum (page 5, lines 52-54).

VERSION 3 - REVIEW

**Reviewer**	**1**
**Name**	**Seo, Songwon**
**Affiliation**	**Korea Institute of Radiological and Medical Sciences**
**Date**	**23-Feb-2026**
**COI**	

The authors have adequately addressed the previous comments, and I have no further major concerns.
